# Sample Limited Characterization of a Novel Disulfide-Rich Venom Peptide Toxin from Terebrid Marine Snail *Terebra variegata*

**DOI:** 10.1371/journal.pone.0094122

**Published:** 2014-04-08

**Authors:** Prachi Anand, Alexandre Grigoryan, Mohammed H. Bhuiyan, Beatrix Ueberheide, Victoria Russell, Jose Quinoñez, Patrick Moy, Brian T. Chait, Sébastien F. Poget, Mandë Holford

**Affiliations:** 1 Department of Chemistry and Biochemistry, City University of New York- Hunter College and Graduate Center, New York, New York, United States of America; 2 The American Museum of Natural History, New York, New York, United States of America; 3 Department of Chemistry, College of Staten Island and Graduate Center, City University of New York, Staten Island, New York, United States of America; 4 NYU Langone Medical Center, New York University, New York, New York, United States of America; 5 The Rockefeller University, New York, New York, United States of America; Instituto Butantan, Brazil

## Abstract

Disulfide-rich peptide toxins found in the secretions of venomous organisms such as snakes, spiders, scorpions, leeches, and marine snails are highly efficient and effective tools for novel therapeutic drug development. Venom peptide toxins have been used extensively to characterize ion channels in the nervous system and platelet aggregation in haemostatic systems. A significant hurdle in characterizing disulfide-rich peptide toxins from venomous animals is obtaining significant quantities needed for sequence and structural analyses. Presented here is a strategy for the structural characterization of venom peptide toxins from sample limited (4 ng) specimens via direct mass spectrometry sequencing, chemical synthesis and NMR structure elucidation. Using this integrated approach, venom peptide Tv1 from *Terebra variegata* was discovered. Tv1 displays a unique fold not witnessed in prior snail neuropeptides. The novel structural features found for Tv1 suggest that the terebrid pool of peptide toxins may target different neuronal agents with varying specificities compared to previously characterized snail neuropeptides.

## Introduction

**Figure 1:**
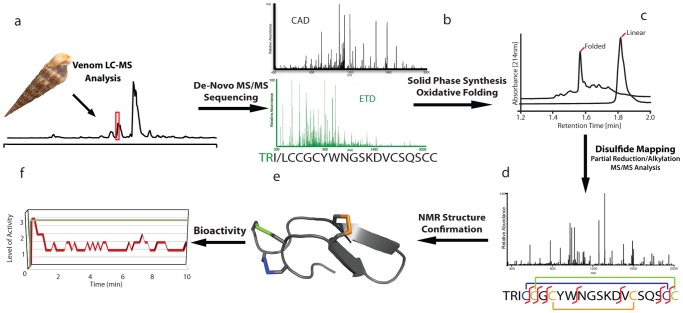
Discovery and characterization of disulfide–rich Tv1 teretoxin. An integrated approach for characterizing sample limited disulfide peptidic natural products was applied: a) RP-HPLC on-line separation of *Terebra variegata* venom (highlighted is the region where the sequenced peptide eluted). b) ETD and CAD MS/MS analysis recorded on the native peptide to determine the sequence of Tv1 peptide. c) RP-HPLC of linear and folded versions of chemically synthesized Tv1 peptide. d) MS/MS spectrum of partially reduced and alkylated Tv1 used to determine the disulfide connectivity. e) NMR solution structure of folded Tv1 peptide. f) Bioactivity of 20 μM of Tv1 (−) versus normal saline (NS) (−) solution injected into polychaete worms. Y-axis indicates level of activity, where 3 is the normal activity of the NS injected worm, 1 and 2 indicate decreased activity due to partial paralysis.

There is a need to discover new compounds that enhance the drug pipeline for treating ailments. The disulfide-rich peptide toxins found in venomous organisms such as snakes, spiders, scorpions, leeches, and marine snails are highly efficient and effective for manipulating physiological pathways and for novel therapeutic and insecticide drug development [1]–[4]. In an effort to identify novel disulfide compounds from venomous snails, we have investigated the Terebridae, a sister group to the more familiar cone snails. Novel peptide toxins from the Terebridae would enhance the pool of therapeutic compounds available for biomedical research. Venomous disulfide-rich compounds include ion channel and enzyme inhibitors, growth factors, and structural or ligand-binding domains [5]. The disulfide-rich peptide library produced by venomous organisms is estimated to contain well over 2 million compounds. The potential and promise of these compounds are evidenced by breakthroughs such as, angiotensin-converting enzyme (ACE)-inhibitor, Captopril [6] and more recently, ziconotide (Prialt), the first drug from a venomous marine snail, *Conus magus*, which is used to treat chronic pain in HIV and cancer patients [7]. Peptides, while currently not as bio-available as small molecule therapeutics, have advantages of higher target specificity and selectivity, and decreased toxicity. Despite their advantages, the use of peptides in drug development lags in the discovery and characterization stages. Obtaining workable quantities of venomous natural products has remained a significant challenge due to the large amount of material that has traditionally been needed to identify and structurally characterize peptidic compounds [8]–[12]. A sensitive and robust method for discovery and characterization of disulfide-rich peptides from sample limited venom sources would facilitate their application as biochemical tools and potential drug development targets [13]–[15]. Presented here is an integrated approach of liquid chromatography mass spectrometric sequencing, chemical peptide synthesis, and NMR for the rapid identification and characterization of sample limited bioactive disulfide-rich venomous snail peptide toxins (Fig. 1). Structural characterization of *Terebra variegata* peptide toxin Tv1 was accomplished using a total venom sample of 4 ng, an amount that would previously make characterization of venom components almost impossible [16], [17].

Tv1 is the first peptide structurally characterized from a terebrid snail. Terebrids are part of the Conoidean superfamily of predatory mollusks, which includes cone snails and turrids [18], [19]. Tv1 is a novel twenty-one amino acid teretoxin peptide with a cysteine scaffold similar to the M-superfamily of cone snail neurotoxins, CC-C-C-CC. Apart from the cysteine pattern, there is no sequence homology between Tv1, TR(I/L)CCGCYWNGSKDVCSQSCC, and known M superfamily conotoxins from *Conus* snails (Table 1). Conoidean venoms are a rich source of neuroactive disulfide-rich peptides, used to investigate cellular communication in the nervous system [17]–[22] and have diverse biomedical applications, including pain therapy [23], [24]. In contrast to conotoxins, terebrid toxins, teretoxins, which were recently identified, [25], [26] are an untapped resource for discovering novel neuropeptides. The sequence and structural characterization approach presented here significantly reduces the number of snails needed, while allowing for rapid characterization of venom components, in an effort to conserve natural resources (Fig. 1).

**Table 1:** Comparison of Teretoxin Tv1 and known M superfamily Conotoxins.

Teretoxin	Sequence	Target(s)	Reference
Tv1	TRI**CC**G**C**YWNGSKDV**C**SQS**CC**	unknown	This work
**Conotoxin**			
MrIIIe	V**CC**PFGG**C**HEL**C**Y**CC**D*	unknown	[23], [33], [49]
TxIIIa	**CC**SWDV**C**DHPS**C**T**CC**G*	unknown	[50], [51]
MrIIIa	G**CC**GSFA**C**RFG**C**VO**CC**V	unknown	[50]
TxIIIb	**CC**PPVA**C**NMG**C**KP**CC** *	unknown	[31], [50]
Reg12a	G**CC**OOQW**C**GOD**C**TSO**CC**	unknown	[52]
Tx3f	R**CC**KFP**C**PDS**C**RYLCC*	unknown	[31]
Qc3.1	A**CC**DPDW**C**DAG**C**YDG**CC**	unknown	[23]
BuIIIA	VTDR**CC**KGKRE**C**GRW**C**RDHSRCC*	Na_v_1.2,1.3, 1.4	[53]
BuIIIB	VGER**CC**KNGKRG**C**GRW**C**RDHSRCC*	Na_v_1.2,1.3,1.4,1.5,1.7	[53]
BuIIIC	IVDR**CC**NKGNGKRG**C**SRW**C**RDHSRCC**	Na_v_1.2,1.3,1.4,1.7	[53]
MIIIA	ZG**CC**NVPNG**C**SGRW**C**RDHAQCC*	TTX-r	[54]
KIIIA	**CC**N**C**SSKW**C**RDHSRCC*	TTX-r	[55]
PIIIA	ZRL**CC**GFOKS**C**RSRQ**C**KOHRCC*	Na_v_1.4	[38]
GIIIA	RD**CC**TOOKK**C**KDRQ**C**K-OQR**CC**A*	Na_v_1.2, 1.4, 1.6–1.8	[56]
GIIIB	RD**CC**TOORK**C**KDRR**C**K-OMK**CC**A*	TTX-r	[56]
GIIIC	RD**CC**TOOKK**C**KDRQ**C**K-OLK**CC**A*	TTX-r	[56]
CnIIIA	GR**CC**DVPNA**C**SGRW**C**RDHAQCC*	TTX-r	[54]
CnIIIB	ZG**CC**GEPNL**C**FTRW**C**RNNAR**CC**RQQ	TTX-r	[54]
SmIIIA	ZR**CC**NGRRG**C**SSRW**C**RDHSR**CC**	TTX-r, TTX-s	[57]
SIIIA	ZN**CC**NGG**C**SSKW**C**RDHARCC*	TTX-r, TTX-s	[58]
CIIIA	GR**CC**EGPNG**C**SSRW**C**KDHARCC*	TTX-r	[59]

## Results and Discussion

### Venom Extraction and De-Novo Sequencing of Tv1

The primary sequence characterization of Tv1 was accomplished using charge-enhanced Electron Transfer Dissociation (ETD) in combination with traditional Collisional Activated Dissociation (CAD) on reduced and alkylated *Terebra variegata* venom [27]. The peptide toxins in the crude venom were reduced and subsequently alkylated using either iodoacetamide or N, N-dimethyl-2-chloro-ethylamine [27]. The latter converts every cysteine residue into a dimethyl lysine analogue, which increases the charge state of each cysteine-rich peptide toxin, resulting in excellent sequence coverage with ETD.

Combining complementary dissociation strategies CAD and ETD facilitates *de novo* sequencing, as each dissociation strategy has specific fragmentation characteristics, which can be used to confirm the corresponding ETD and CAD spectra (Fig. 1). This is advantageous as peptide toxin sequences can be obtained directly from crude venom without prior fractionation and using only mass spectrometry [27]. This feature is particularly important for sequencing peptide toxins from animals producing minimal amounts of venom, such as terebrids, where only nanogram quantities are extracted from each snail specimen. Four nanograms of crude *Terebra variegata* salivary gland contents were extracted with 30% acetonitrile/water in 0.1% trifluoroacetic acid. An aliquot of the crude venom was analyzed using LC-MS on an Orbitrap XL mass spectrometer to determine the number of teretoxins present. LC-MS analysis was repeated after reduction of the crude venom to determine the number of cysteine residues present in each component. At this point, Tv1 was selected for further analysis due to its similarity in molecular weight and number of cysteines to M superfamily conotoxins. A reduced version of Tv1 was dissociated using CAD (Fig. 1b, and Figure S1 in File S1) and the dimethyl lysine analogue version of the teretoxin was dissociated using ETD (Fig. 1b and Figure S2 in File S1). The ETD MS/MS spectrum resulted in almost 100% sequence coverage. The sequence assignment by ETD could be supported with the CAD spectrum on the reduced toxin (Fig. 1b and Figure S1 in File S1). There were two places of ambiguity in the Tv1 peptide sequence: (1) the inherent uncertainty of isoleucine (Ile) and leucine (Leu) at the third position, which cannot be differentiated with the mass spectrometer used for this analysis, and (2) the order of the first two amino acids, Thr, Arg or Arg, Thr. The second ambiguity was resolved based on the absence of the c1 ion, and the resulting Tv1 sequence was determined as TR(I/L)CCGCYWNGSKDVCSQSCC. However, the Ile/Leu ambiguity at position 3 could not be resolved as there was not sufficient crude extract remaining after MS characterization.

**Figure 2:**
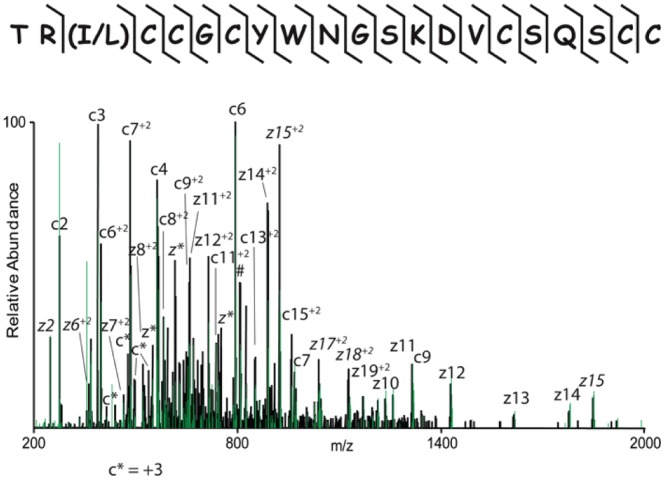
Sequence identification of Tv1 teretoxin. Shown is the native toxin (black) and the synthesized toxin (green) MS/MS spectrum recorded on a (M +6H)+6 ion after conversion of cysteine residues to dimethyl lysine analogs. Note the overlap of all major fragment ions. The sequence is given above the spectrum and observed c and z-type fragment ions are indicated in the sequence with 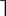 and 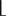, respectively. Doubly charged fragment ions of type c and z are labeled with +2, triply charged ions are of type c and z are indicated with *, z-type fragment ions that resulted from cleavage at cysteine with subsequent loss of the cysteine side chain are denoted in italic [60] and charge reduced species are labeled in the spectrum with #. The spectrum was recorded at a resolution of 7500 at m/z 400 and all fragment ions have a mass accuracy of better than 5 ppm.

To confirm Tv1 *de novo* assignment, the peptide was chemically synthesized using Fmoc Solid Phase Peptide Synthesis (SPPS). Due to inherent uncertainty in the amino acid sequence at position 3, both Ile3 and Leu3 containing peptides were synthesized and subjected to the same chemical derivatization strategy. The two synthesized Tv1 teretoxins both resulted in an MS/MS spectrum identical to the native peptide, confirming the *de novo* sequence assignment in which TR is the order of the first two amino acids, but with remaining I/L uncertainty at position 3 due to the mass spectrometer used (Fig. 2 and Figure S2 in File S1). The novel peptide teretoxin, Tv1 from venomous marine snail *Terebra variegata* was thus identified and its sequence was determined.

### Synthesis, Characterization and Oxidative Folding of Tv1

Tv1 was chemically synthesized using Fmoc Solid Phase Peptide Synthesis without prior knowledge of disulfide connectivity, and all cysteine residues were protected with a standard side chain protecting group, trityl. The synthesized Tv1 peptide was purified to 99% using RP-HPLC (Figure S3 in File S1) and its mass was confirmed by MALDI mass spectrometry. The expected average mass of the linear peptide [M+H] 2316.3 m/z was observed (Figure S4 in File S1). Tv1 was folded using thiol-assisted air oxidation reaction and purified by HPLC (Figure S3 in File S1). The mass of folded Tv1, 2310.7 m/z, is reduced by 6 Da confirming that three intramolecular disulfide bonds had been formed (Figure S5 in File S1). No differences were observed in synthesis and folding kinetics of the two Tv1 peptides with Ile or Leu at the third amino acid position, and both versions of Tv1 were used to determine the disulfide connectivity of the peptide.

### Disulfide Mapping of Tv1

**Figure 3:**
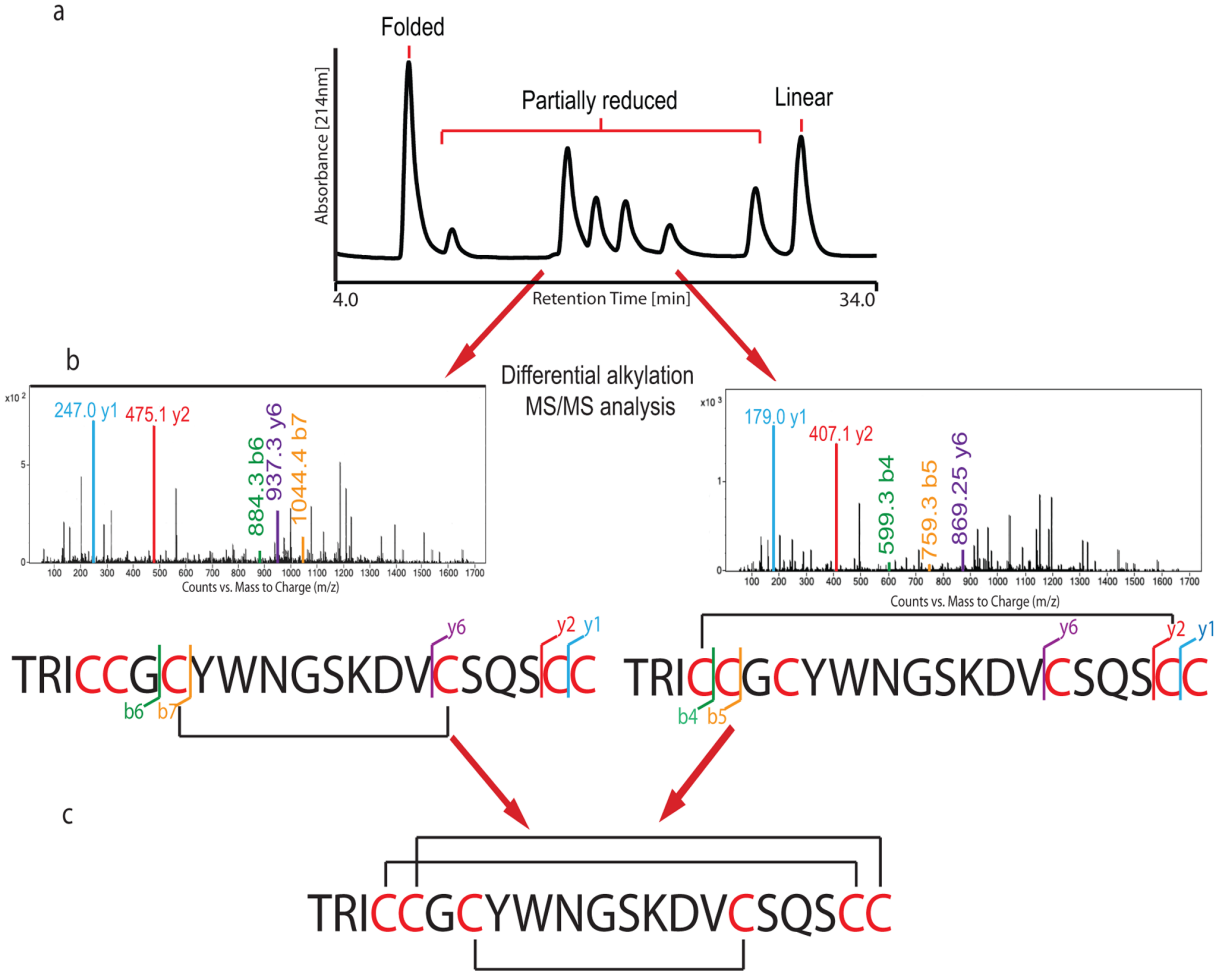
Disulfide mapping of Tv1. a) RP-HPLC fractionation of all differentially folded peaks (on HPLC gradient of 20% to 35% buffer B (80% Acetonitrile, 0.1% TFA) in buffer A (water, 0.1% TFA) over 75 min. b) MS-MS spectrum of partially reduced peptide peaks showing representative alkylated b and y ions. c) Schematic representation of Tv1 with predicted disulfide bridges.

Due to the limited number of enzymatic cleavage sites and the presence of adjacent cysteine residues, traditional strategies for disulfide mapping such as tryptic digestion and acid hydrolysis were difficult to implement. The disulfide connectivity of Tv1 was independently determined by MS/MS mapping and NMR spectroscopy. For MS/MS mapping, a partial reduction and dual alkylation protocol was applied using reducing agent TCEP-HCl (Tris(2carboxyethyl)phosphine hydrochloride) and alkylating agents NEM (N-ethylmaleimide) and IAM (iodoacetamide) [28]–[30]. Chemically synthesized and oxidatively folded Tv1 peptide was first partially reduced at pH 3.0 with TCEP, and subsequently alkylated with NEM. The partially alkylated Tv1-NEM peptide was reduced further with TCEP at pH 7.0 and alkylated again with IAM this time. Dual NEM/IAM alkylation resulted in Tv1 peptide species that were labeled with 2, 4 or 6 NEM and IAM groups (Fig. 3 and Figure S6 in File S1). The location of NEM and IAM modifications in each of the six partially reduced species was determined by matching the MS/MS b- and y- series ions to theoretical patterns. All six partially reduced species provided complementary, non-contradictory information and the resulting connectivity was determined to be Cys4–Cys20, Cys5–Cys21 and Cys7–Cys16 (Fig. 3 and Table S1 in File S1). Tv1 has a cysteine scaffold similar to M Superfamily conotoxins (Table 1), but it displays a disulfide pattern previously unknown in native cone snail peptides [23], [31]–[35] with a disulfide bridge formed between the two middle cysteines (Cys7 and Cys16) and a parallel 2-disulfide bond “staple” (Cys4–Cys20 and Cys5–Cys21) linking the N- and C-termini of the peptide.

### Characterization of Tv1 by NMR Solution Structure Derivation

To confirm the disulfide bond connectivity derived from MS, the solution structure of Tv1 was derived using standard homonuclear proton NMR techniques on unlabeled folded synthetic peptide. Initial NMR experiments were carried out on Tv1 peptide containing either Ile or Leu at the third position. The spectra were very similar for both versions of the peptide, with the only differences found in the signals of the Ile3 and Leu3 residues themselves, indicating that both peptides adopt the same structure. Subsequent NMR structural calculations were carried out using only the Ile3 form of the peptide.

Proton assignments were obtained from 2D NOESY and TOCSY spectra, and carbon chemical shifts were assigned with the help of a natural-abundance ^13^C-HSQC spectrum (Table S2 in File S1). Initial structure calculation runs were carried out using only manually assigned Nuclear Overhauser Effect (NOE) distance restraints. Disulfide connectivities were then determined based on proximity of cysteine residues in the 10 lowest-energy structures and were in agreement with the disulfide bond pattern derived by MS analysis.

Direct evidence for the Cys7–Cys16 disulfide bond was also observed from NOE crosspeaks between the H^N^ of Cys16 and the H^α^ of Cys7, as well as a number of connectivities to the two flanking residues, Gly6 and Tyr8 (Figure S7 in File S1). Once the disulfide linkages were determined, additional distance restraints for the disulfide bonds were introduced. The final round of structure calculations was run with covalent bonds between the connected cysteine side chains. A bundle of the 10 lowest-energy structures was obtained with an RMSD of 0.42 Å for backbone and 0.74 Å for all heavy atoms (Figure S8 and Table S3 in File S1). A query for other peptide structures similar to the determined Tv1 structure using the software packages VAST [36] or PDBeFOLD [37] did not yield any hits, indicating that the structure of Tv1 represents a new fold for small peptide toxins. The most prominent and best-defined structural feature is a β-hairpin from Cys7 to Cys16 that is clamped together by the disulfide bond formed between these two residues (Fig. 1 and Figure S9 in File S1). The remainder of the peptide wraps around the side chain aromatic ring of Tyr8, in particular through the formation of hydrophobic interactions with the side chains of Ile3 and Val15. The N- and C-terminal loops are clamped together in an antiparallel way through the Cys4–Cys20, Cys5–Cys21 double-disulfide bond arrangement, giving the whole teretoxin the shape of a flattened ellipsoid (Figure S8 in File S1). Coordinates for the Tv1 structures have been submitted to the PDB and are available under accession code 2mix.

**Figure 4:**
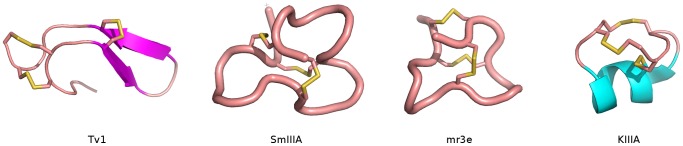
Structural comparison of Tv1 teretoxin and M-superfamily conotoxins. Comparison of the NMR structure of Tv1 with that of M superfamily conotoxins SmIIIA, mr3e, and KIIIA reveals significant structural differences between Tv1 and these conotoxins despite all having the same cysteine scaffold CC-C-C-CC. All structures are shown in cartoon representation with disulfide bonds highlighted in yellow. All figures were prepared using PyMol (www.pymol.org). Conotoxin structural references are as follows: MrIIIe [33] SmIIIA [61] and KIIIA [62].

Although Tv1 has a cysteine scaffold similar to M Superfamily conotoxins (Table 1), the observed differences in fold and disulfide bonding pattern are not surprising given the lack of sequence homology to M superfamily conotoxins. It is interesting to note that the loop between C3 and C4 is at least 4 amino acid residues longer in Tv1 compared to any of the other listed conotoxins, which is in agreement with the fact that these are the residues involved in the formation of the β-hairpin that is lacking in conotoxins. No β-strand structures are found in any of the other M-superfamily conotoxins (Fig. 4) [28]–[32]. To our knowledge, the only other example of Tv1’s disulfide bond pattern was found in an active non-native minor refolding product for μ-conotoxin PIIIA [38]. The 3D structures of both the native and non-natively refolded forms of PIIIA are very different from the structure of Tv1 as they contain segments of α-helix and no β-sheet structure. The difference between Tv1 and the PIIIA refolded product with the same disulfide bridge arrangement further supports the conclusion that Tv1 is representative of a new and unique group of peptide toxins.

### Bioactivity Assay

To determine if synthetic Tv1 is biologically active, the peptide was injected into polychaete worms, which are the natural prey of terebrid snails. Injections of 20 μM Tv1 (Ile) into *N. virens* polychaete worms caused partial paralysis (Fig. 1f, Table 2). This finding is consistent with the recent study where crude terebrid venom extract exhibited nAChR inhibitory activity [39]. Inhibition of nicotinic receptors at the neuromuscular junction can result in paralysis. Further experiments are underway to determine the specific molecular target of Tv1.

**Table 2:** Tv1 bioactivity in polychaete worms.

Activity	% Time spent	
	Tv1 (20 μM)	NSS
at 0	0.995	1.45
Between 0 and 1	74.47	0
Between 1 and 2	19.98	0
Between 2 and 3	4.55	98.55

## Conclusion

A novel teretoxin, Tv1, from *Terebra variegata* was structurally identified and found to be biologically active. The strategy applied to the discovery and characterization of Tv1 allows for the unequivocal analysis of minuscule amounts of sample. Four nanograms of venom sample was used to elucidate the amino acid sequence of Tv1. Such a minute amount of sample would previously be inaccessible for natural product discovery from venomous organisms. There are over 400 different species of terebrids, each predicted to express between 50–200 different peptide toxins in their venom. With an integrated approach of mass spectrometry, chemical peptide synthesis, and NMR as applied to Tv1, it will be possible to access the substantial quantity of disulfide-rich peptides (>80,000) in the Terebridae. The novel structural features of Tv1 suggest that teretoxins will reveal different mechanisms of action and different target specificities compared to disulfide-rich peptide toxins previously characterized and used as tools in neuroscience and as drug leads. In addition, an abundance of gastropods, including most species of the venomous marine conoidean snails, are smaller than 8 mm in shell size [40], highlighting the need for an efficient microscale mass spectrometry sequencing strategy. The sample limited integrated approach presented here can significantly enhance discovery and characterization of the vast disulfide-rich natural product peptide libraries produced by various venomous organisms.

## Methods

### Venom Extraction and De-Novo Sequencing

Four *Terebra variegata* snails were collected by MH from the Las Perlas Archipelago in Panama on an expedition with the RV URRACA and glands were dissected. Scientific research permits were provided by the Smithsonian Tropical Research Institute Permit Office (STRI-SPO) and The Panama Aquatic Resources Authority (ARAP). Pooled crude venom contents from all 4 specimens were extracted with aid of light sonication in 30% acetonitrile/water acidified with 0.1% trifluoroacetic acid and centrifuged. The supernatant was lyophilized and stored at −20°C. An aliquot of crude venom was pressure loaded onto a self-packed PicoFrit column (New Objective) with integrated emitter tip (360 μm o.d., 75 μm i.d., 15 μm tip), packed with 6 cm of reverse-phase C18 material (Alltima C18 5 μm beads, Alltech Associates), rinsed for 10 min with 0.1 M acetic acid and subsequently gradient eluted with a linear gradient from 0 to 100% B in 30 min (A = 0.1 M acetic acid, B = 70% acetonitrile in 0.1 M acetic acid, flow rate 120 nL/min) into an LTQ Orbitrap XL with ETD (ThermoFisher Scientific), using a home-built micro electrospray source with a liquid junction.

The instrument was operated in data dependent mode cycling through full scan (300–2,000 m/z, single scan) followed by 8 MS/MS scans on the 8 most abundant ions from the immediate preceding full scan. Next, aliquots of reduced and iodoacetamide alkylated crude venom were pressure loaded onto a self-packed PicoFrit column (New Objective) with integrated emitter tip as described above and eluted using the same gradient as above into the LTQ Orbitrap XL with ETD. Instrument was operated in a data dependent mode cycling through a full scan (300–2,000 m/z, single scan) followed by 8 MS/MS scans on the 4 most abundant ions from immediate preceding full scan. The instrument was programmed to acquire first 4 CAD and then 4 ETD spectra on the 4 most abundant ions. The cations were isolated with a 2-Da mass window and set on a dynamic exclusion list for 30 seconds after they were first selected for MS/MS. Target value for MS/MS was set to 10^4^ cations and 10^6^ anions of fluoranthene. For CAD an activation time of 30 ms was chosen. For ETD the ion/ion reaction time was set to 100 ms. In both cases 4 scans were averaged per MS/MS spectrum. In a majority of cases, complete sequence elucidation after this initial analysis was not possible, but based on this initial screen optimal charge states for subsequent targeted ETD analysis were chosen. For subsequent analysis either reduced, iodoacteamide alkylated, or dimethyl lysine analog versions of toxin were pressure loaded onto the self-packed columns as described above. The column was rinsed for 10 min (for reduced or iodoacetamide alkylated toxins) or for 20 min (for lysine dimethyl analog versions of toxins) with 0.1 M acetic acid and gradient eluted into the LTQ XL as described above. To obtain higher quality MS/MS spectra in this second analysis, 12 scans were averaged and the data acquired in high resolution with 7,500 resolution setting. The spectra were manually interpreted (Figures S1 and S2 in File S1).

### Synthesis and Purification of Tv1

Tv1 peptide was synthesized by microwave assisted Fmoc SPPS on a CEM Liberty synthesizer using standard side chain protection. Standard Fmoc cysteine with side chain protecting group Trityl (Fmoc-Cys (Trt)-OH) was used for all cysteine residues in the Tv1 peptide. Following treatment of peptidyl resin with Reagent K [92.5% TFA (Trifluoroacetic acid), 2.5% TIS (Triisopropylsilane), 2.5% EDT (1,2 Ethanedithiol) and 2.5% water, 4 hr)] and cold ether precipitation, crude Tv1 was purified by RP-HPLC using an X-Bridge semipreparative column (10×150 mm, 5 μm particle size, Waters Corporation, Milford, MA, USA). Elution was carried out at 5 mL/min with 20% buffer B (80% Acetonitrile, 0.1% TFA) and 80% buffer A (0.1% TFA) for the first 5 min, then increasing buffer B to 35% in 45 min. HPLC buffer composition remained the same all through the work. Purity of collected peptide was confirmed by RP-UHPLC using an Acquity UPLC (BEH 300 C18 1.7 μm, Waters Corporation) column and eluted using a linear gradient from 0% to 75% buffer B in 3.5 min (Figure S3 in File S1). The identity of synthesized peptide was confirmed by molecular mass measurement of purified peptide using MALDI-TOF (Waters, Micromass, CHαCN matrix) (Figure S4 in File S1).

### Oxidative Folding of Tv1

A one-step thiol-assisted oxidation was used to prepare folded Tv1 peptide. The linear peptide (20 μM) was incubated in 0.1 M Tris –HCl, 0.1 M NaCl, 100 μM EDTA, 1 mM GSH, 1 mM GSSG, pH 7.5. The folding reaction was terminated by acidification with 8% formic acid at 15 min, 30 min, 1, 2, 3, 4, and 24 h and the folding yield monitored using UHPLC. A preparative scale folding reaction was then conducted at an optimized time of 2 h, and the folded peptide was purified using X-Bridge semipreparative column (as mentioned earlier). Elution was carried out at 5 mL/min with 15% buffer B and 85% buffer A for the first 5 min, then increasing buffer B to 35% in 45 min. The purity was confirmed using Acquity UHPLC column (Figure S3 in File S1), and the molecular mass of the oxidized peptide was confirmed by MALDI-TOF (Figure S5 in File S1).

### Disulfide Mapping by Tandem MS/MS

A partial reduction and dual alkylation strategy was employed using reducing agent TCEP (Tris carboxy ethyl-phosphine hydrochloride, Thermo-Fisher) and alkylating agents NEM (N-ethylmaleimide, Sigma) and IAM (iodoacetamide, Sigma). Conditions for the partial reduction and alkylation of folded peptides were optimized modifying previous protocols [30], [41]. Briefly, 9 μl of folded peptide (1 mM) was dissolved in 0.1 M citrate buffer, pH 3.0 and 1 μl of TCEP (20 mM in 0.1 M citrate buffer, pH 3.0) was added to it. Reduction was carried out at 4°C for 90 min and was terminated by injecting onto an RP-HPLC column [X-Bridge analytical column (5×150 mm, 5 μm particle size, Waters Corporation, Milford, MA, USA)] and eluted with 20% buffer B in buffer A for 5 min, then increasing buffer B to 35% in 75 min. Six partially reduced peaks (Figure S6 in File S1) were each collected directly into microcentrifuge tubes containing 100 μl NEM (200 mM in 0.1 M citrate buffer pH 3) and incubated at 37°C for 2 h in the dark. Partially reduced and alkylated reactions were then lyophilized and desalted to remove unreacted NEM using UHPLC. The desalted fractions were incubated with 100 mM TCEP at 55°C for 1 h to completely reduce the NEM labeled peptide and alkylated with 275 μM IAM for 2 h at 37°C in dark. The alkylation steps resulted in peptide species that were labeled with 2, 4 or 6 NEM or IAM groups. The labeled lyophilized species were suspended in 0.5% formic acid and subjected to LC-MS/MS analysis on 43 mm HPLC-Chip/Q-TOF using a 7 min gradient to resolve fully labeled species from those with incomplete alkylations. Data was collected in targeted and auto MS/MS mode and processed using molecular feature extraction (MFE) software to detect unique peptide features (MS and MS/MS spectra) followed by sequence matching on MassHunter Bioconfirm Qual B.05 software (Table S1 in File S1).

### NMR Solution Structure Derivation

Samples for Nuclear Magnetic Resonance (NMR) studies were prepared by dissolving lyophilized oxidized peptide into either a 9∶1 ratio of H_2_O:D_2_O or in 100% D_2_O at a concentration of ∼260 μM. Sample pH was adjusted to pH 6.0 and 50 μM trimethylsilyl propanoic acid was added as a reference compound. Spectral acquisition was carried out at 5°C on a Varian Inova 600 MHz NMR spectrometer equipped with a cryogenically cooled HCN probe. 2D-TOCSY spectra were acquired in 90% H_2_O (64 scans with 1024×512 points) and D_2_O (48 scans with 1024×512 points) with spin-lock times of 60 ms, whereas 2D-NOESY spectra were acquired with mixing times of 200 ms in H_2_O with a matrix size of 1024×512 points in 64 scans and in D_2_O with a similar matrix size in 112 scans. A natural abundance ^1^H^13^C HSQC correlation spectrum was also acquired in 1024 scans with a matrix size of 1024×80 points. Acquired spectra were processed with NMRPipe [42] and analyzed with CCPNmr Analysis [43]. Proton assignments were made by comparison of TOCSY and NOESY spectra, and used to assign the ^13^C chemical shifts through the HSQC spectrum (Table S2 in File S1). Distance restraints for structural calculations were obtained from 2 NOESY spectra: one spectrum collected in 90% H_2_O with a mixing time of 200 ms and another spectrum collected in D_2_O with a mixing time of 400 ms. Manual assignment was performed for about 90% of all NOE cross-peaks. Dihedral angle restraints were derived from the assigned chemical shifts using the software TALOS+ [44]. Structural calculations were carried out with ARIA2 [45]/CNS [46]. Initial structural calculations were conducted using only the manual NOE assignments (with distances automatically calibrated by the software based on peak volumes) and without any restraints for the disulfide bonds. An ARIA2 run consisting of 8 iterations of simulated annealing followed by explicit water refinement of the 10 lowest-energy structures was performed. Disulfide connectivities were determined based on this initial structural bundle and included in the final round of structural calculations as covalent bonds. Eight iterations were performed in ARIA with 20 structures generated per iteration and the 7 lowest energy structures of each iteration forming the structural ensemble for the next iteration. For this run, NOE assignments were allowed to be automatically adjusted by the software during the calculation. For the last iteration, 100 structures were generated, and ten lowest-energy solutions were subjected to explicit solvent refinement. The quality of the final structural bundle was assessed by Procheck NMR (Table S3 in File S1) [47].

### Bioactivity Assay on Polychaete Worms

*Nereis virens* (polychaete worms) (<3.5 g) were maintained at 4°C in salt water prior to injection. Worms were laid on a Styrofoam block and injected with an insulin syringe. A modified needle cap was used to maintain a consistent 1.5 mm depth of needle puncture. Worms were injected between the 5^th^ and 7^th^ segments on the ventral anterior end with the aim of targeting the ventral nerve cord. Control worms were injected with 12 μl of 0.9% normal saline solution (NSS). Lyopholized samples of Tv1 toxin were resuspended in NSS and diluted to a concentration of 20 μM which was confirmed using a ThermoScientific Nanodrop 2000c spectrophotometer. At least three worms were used for each trial- one saline control and two injected with Tv1 toxin for a total of 3 trials. After injection, the worms were transferred to side-by-side containers filled with room temperature salt water. The worm movements were then recorded by video in order to evaluate the effects of the toxin over a period of at least 3.5 hours. Varying temperature elicits different phenotypic responses from *N. virens*: water at 4°C has an anesthetizing effect, while room temperature prompts a vigorous response [48].

Taking the worms from 4°C water and immersing them in room temperature water (25°C) immediately after injection stimulates an active response from the worms, making it easier to recognize paralytic (or partially paralytic) effects induced by a novel toxin in the first minutes after injection. Worms were observed every other day for two weeks after injection. The recorded videos were analyzed blind by two independent observers; the activity of each worm over a small time interval was noted on a scale of 1–3, with 3 being extremely active and 1 being inactive. Each video was analyzed and annotated three times by each observer. Observations for each worm began at the time of injection (Table S4 in File S1). The numerical results of the two independent viewings were averaged together if results were similar, if two viewers disagreed, a third observer was employed to review the disputed section of video. Results of relative activity vs. time were plotted to quantify the phenotypic effects of the toxin and saline injection on *N. virens* (Fig. 1f). The data was also used to calculate what percentage of time each worm spent inactive (a rating of 1), active (an averaged rating of 1.5–2), and extremely active (rating 2.5–3) (Table 2).

## Supporting Information

File S1
**This file contains Figure S1–Figure S9 and Table S1–Table S4.** Figure S1, CAD of native Tv1. MS/MS spectrum recorded on a (M +2H)^+2^ ion after reduction of cysteine residues. The sequence is given above the spectrum and observed b, a and y-type fragment ions are labeled in the spectrum. Observed peptide backbone cleavage is indicated in the sequence above with 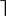 and 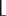 for N- and C-terminal fragment ions, respectively. Doubly charged fragment ions are labeled with ^+2^. The neutral loss of water from the precursor ion is shown as [M+2H]^+2^–H_2_0, but neutral losses of fragment ions are not labeled. The spectrum was recorded at a resolution of 7500 at m/z 400 and all fragment ions have a mass accuracy of better than 5 ppm. Figure S2, ETD of native (black) and synthetic Tv1 (blue). MS/MS spectrum recorded on a (M +6H)^+6^ ion after conversion of cysteine residues to dimethyl lysine analogs. The sequence is given above the spectrum and observed c and z-type fragment ions are indicated in the sequence with 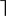 and 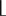, respectively. Doubly charged fragment ions of type c and z⋅ are labeled with ^+2^, triply charged ions are of type c and z are indicated with *, z-type fragment ions that resulted from cleavage at cysteine with subsequent loss of the cysteine side chain are denoted in *italic* and charge reduced species are labeled in the spectrum with #. The spectrum was recorded at a resolution of 7500 at m/z 400 and all fragment ions have a mass accuracy of better than 5 ppm. Figure S3, RP-UHPLC chromatograms of Tv1 linear and oxidized peptide at 214nm. During a pilot folding reaction, over 90% of the linear Tv1 peptide fully oxidized and showed a peak at 1.58 minute in comparison to 1.83 minute linear Tv1 peak at the gradient of 0–75% buffer B (80% acetonitrile, 0.1% TFA) in buffer A (0.1% TFA) within two hours. Figure S4, Analysis of linear Tv1 peptide by MALDI-TOF mass spectrometry. MALDI-TOF spectrum of Tv1 peptide using α-Cyano-4-*hydroxycinnamic acid* matrix. Figure S5, Analysis of oxidized Tv1 peptide by MALDI-TOF mass spectrometry. MALDI-TOF spectrum of Tv1 peptide using α-Cyano-4-*hydroxycinnamic acid* matrix. Figure S6, RP-UHPLC analysis of partially reduced Tv1 peptide. On a UPLC (BEH 300 C18 1.7 μm, Waters Corporation, Milford, MA, USA) column, a linear gradient of 0–75% buffer B (80% acetonitrile, 0.1% TFA) in buffer A (0.1% TFA) over 6 minutes and peaks were assigned by their degree of reduction. Labels indicate how many disulfides are present. Figure S7, NOE contacts confirming the C7–C16 disulfide bond. An overlay of the H^N^H^α^ fingerprint region of the NOESY (in black) and TOCSY (in blue) spectra shows NOE crosspeaks linking Cys 16 and Cys 7 as well as contacts in the residues flanking the C7–C16 disulfide bond (C7-S17, Y8-C16, G6-C16, G6-N18). Figure S8, Bundle of the 10 lowest energy structures of Tv1 after explicit water refinement. The 10 lowest energy structures are shown in stick representation, displaying the tight convergence found in the final structural bundle. Figure S9, Structure of Tv1. An overlay of a cartoon representation and a stick model of the lowest-energy structure of Tv1 shows the β-sheet character of the peptide and reveals the important role of the Tyr 8 side chain in the formation of a small hydrophobic core. Table S1, Predicted and observed of b and y ions of differentially alkylated peptides by auto and targeted MS/MS analysis. Table S2, Chemical shift assignments of Tv1 (in ppm). Table S3, Structural statistics for the final 10 models of Tv1. Table S4, Sample raw data of Tv1 bioactivity in polychaete worms.(DOC)
