# Supplementary material for: Sample Limited Characterization of a Novel Disulfide-Rich Venom Peptide Toxin from Terebrid Marine Snail Terebra variegata
Source: PLoS One. 2014 Apr 8;9(4):e94122. doi: 10.1371/journal.pone.0094122 (PMC3979744; doi:10.1371/journal.pone.0094122)
Supplement: File S1 — This file contains Figure S1–Figure S9 and Table S1–Table S4. Figure S1, CAD of native Tv1. MS/MS spectrum recorded on a (M +2H)+2 ion after reduction of cysteine residues. The sequence is given above the spectrum and observed b, a and y-type fragment ions are labeled in the spectrum. Observed peptide backbone cleavage is indicated in the sequence above with and for N- and C-terminal fragment ions, respectively. Doubly charged fragment ions are labeled with +2. The neutral loss of water from the precursor ion is shown as [M+2H]+2–H20, but neutral losses of fragment ions are not labeled. The spectrum was recorded at a resolution of 7500 at m/z 400 and all fragment ions have a mass accuracy of better than 5 ppm. Figure S2, ETD of native (black) and synthetic Tv1 (blue). MS/MS spectrum recorded on a (M +6H)+6 ion after conversion of cysteine residues to dimethyl lysine analogs. The sequence is given above the spectrum and observed c and z-type fragment ions are indicated in the sequence with and , respectively. Doubly charged fragment ions of type c and z⋅ are labeled with +2, triply charged ions are of type c and z are indicated with *, z-type fragment ions that resulted from cleavage at cysteine with subsequent loss of the cysteine side chain are denoted in italic and charge reduced species are labeled in the spectrum with #. The spectrum was recorded at a resolution of 7500 at m/z 400 and all fragment ions have a mass accuracy of better than 5 ppm. Figure S3, RP-UHPLC chromatograms of Tv1 linear and oxidized peptide at 214nm. During a pilot folding reaction, over 90% of the linear Tv1 peptide fully oxidized and showed a peak at 1.58 minute in comparison to 1.83 minute linear Tv1 peak at the gradient of 0–75% buffer B (80% acetonitrile, 0.1% TFA) in buffer A (0.1% TFA) within two hours. Figure S4, Analysis of linear Tv1 peptide by MALDI-TOF mass spectrometry. MALDI-TOF spectrum of Tv1 peptide using α-Cyano-4-hydroxycinnamic acid matrix. Figure S5, Analysis of oxidize [file pone.0094122.s001.doc]

**Supporting Information**

**Sample limited characterization of a novel disulfide-rich venom peptide toxin from terebrid marine snail Terebra variegata**

Prachi Anand^1^, Alexandre Grigoryan^1^, Mohammed H. Bhuiyan^3^, Beatrix Ueberheide^4^, Victoria Russell^1^, Jose Quinoñez^1^, Patrick Moy^1^, Brian T. Chait^5^, Sébastien F. Poget^3^, Mandë Holford*^1, 2^

^1^Department of Chemistry and Biochemistry, City University of New York- Hunter College and Graduate Center, New York, New York, USA

^2^The American Museum of Natural History, New York, New York, USA

^3^Department of Chemistry, College of Staten Island and Graduate Center, City University of New York, Staten Island, New York, USA

^4^NYU Langone Medical Center, New York University, New York, New York, USA

^5^The Rockefeller University, New York, New York, USA)

Supplementary Figures (9 figures)

Supplementary Tables (4 tables)

***Supplementary Figures***


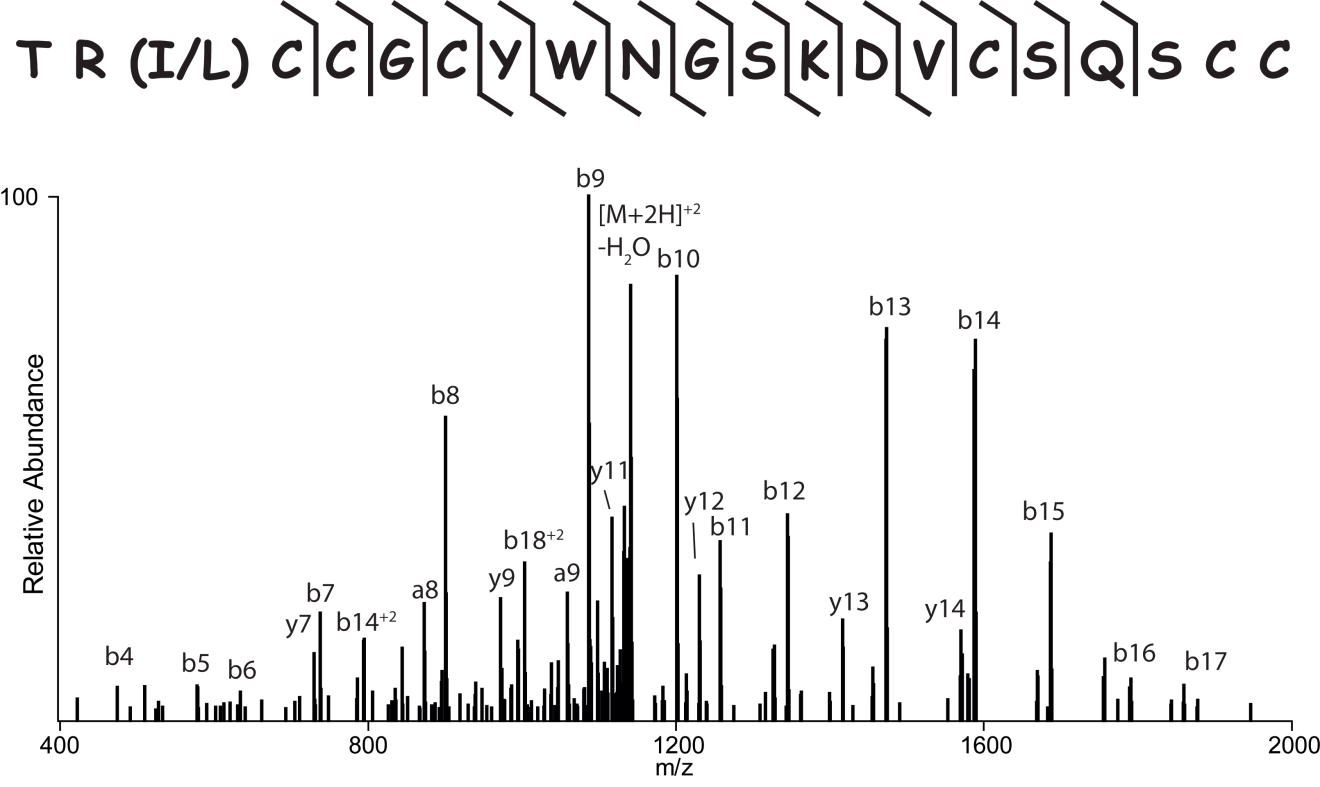


**Figure S1. CAD of native Tv1.** MS/MS spectrum recorded on a (M + 2H)^+2^ ion after reduction of cysteine residues. The sequence is given above the spectrum and observed b, a and y-type fragment ions are labeled in the spectrum. Observed peptide backbone cleavage is indicated in the sequence above with  and  for N- and C-terminal fragment ions, respectively. Doubly charged fragment ions are labeled with ^+2^. The neutral loss of water from the precursor ion is shown as [M+2H]^+2^ –H_2_0, but neutral losses of fragment ions are not labeled. The spectrum was recorded at a resolution of 7500 at m/z 400 and all fragment ions have a mass accuracy of better than 5 ppm.


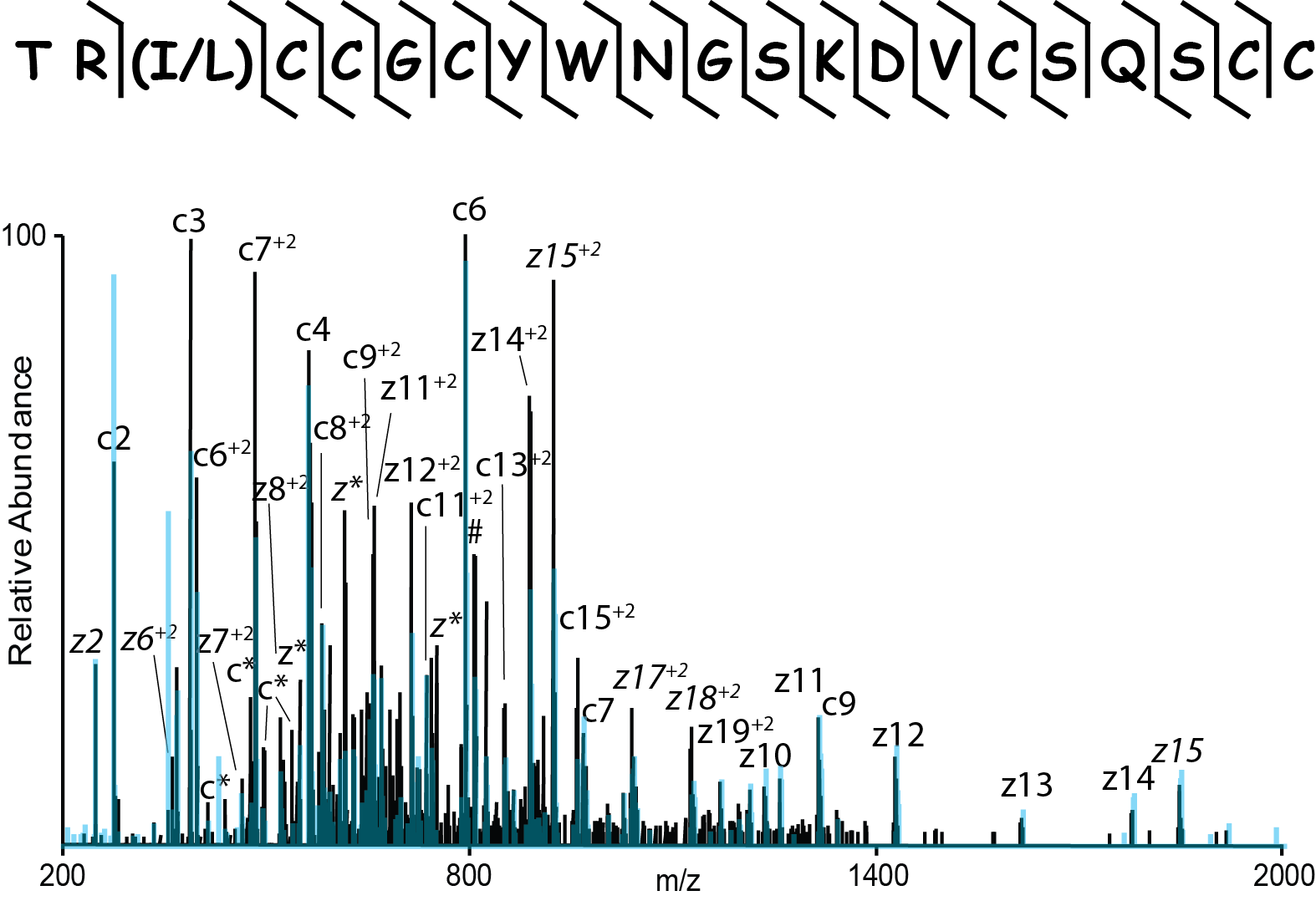


**Figure S2. ETD of native (black) and synthetic Tv1 (blue).** MS/MS spectrum recorded on a (M + 6H)^+6^ ion after conversion of cysteine residues to dimethyl lysine analogs. The sequence is given above the spectrum and observed c and z-type fragment ions are indicated in the sequence with  and , respectively. Doubly charged fragment ions of type c and z⋅ are labeled with ^+2^, triply charged ions are of type c and z are indicated with *, z-type fragment ions that resulted from cleavage at cysteine with subsequent loss of the cysteine side chain are denoted in *italic*^[1]^ and charge reduced species are labeled in the spectrum with #. The spectrum was recorded at a resolution of 7500 at m/z 400 and all fragment ions have a mass accuracy of better than 5 ppm.


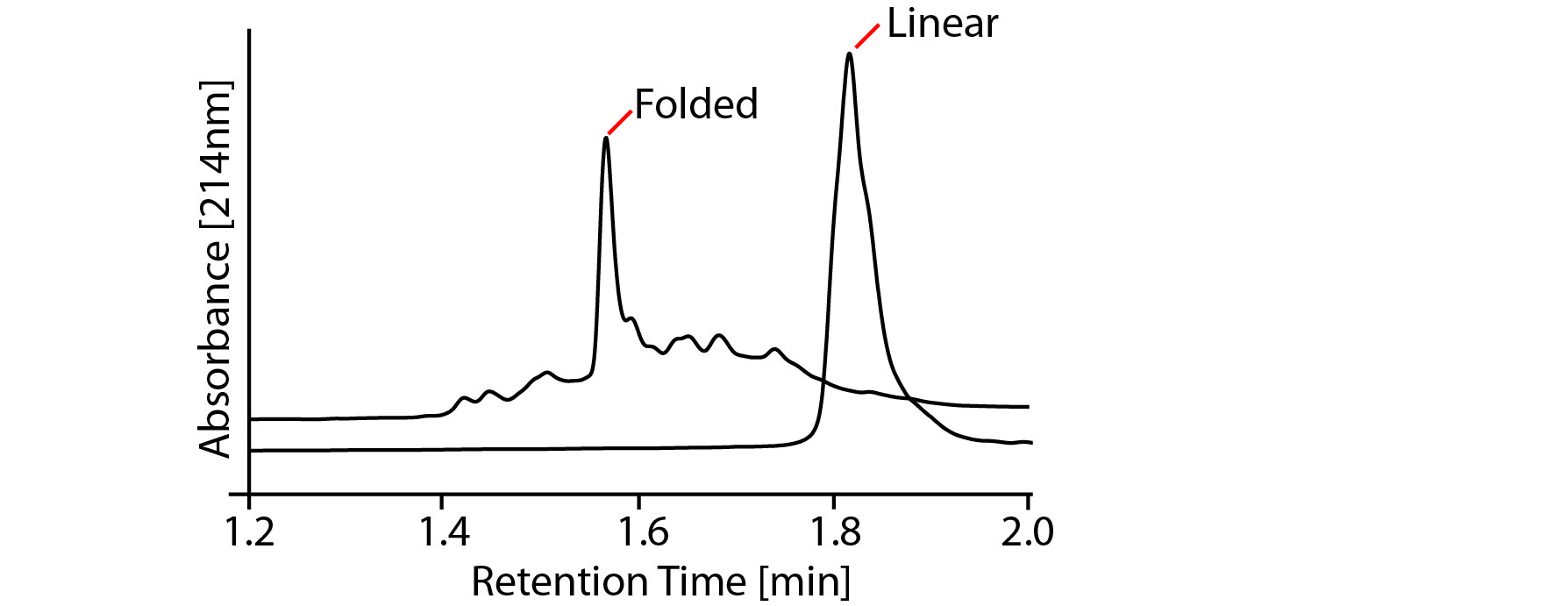


**Figure S3. RP-UHPLC chromatograms of Tv1 linear and oxidized peptide at 214nm.** During a pilot folding reaction, over 90% of the linear Tv1 peptide fully oxidized and showed a peak at 1.58 minute in comparison to 1.83 minute linear Tv1 peak at the gradient of 0-75% buffer B (80% acetonitrile, 0.1% TFA) in buffer A (0.1% TFA) within two hours.


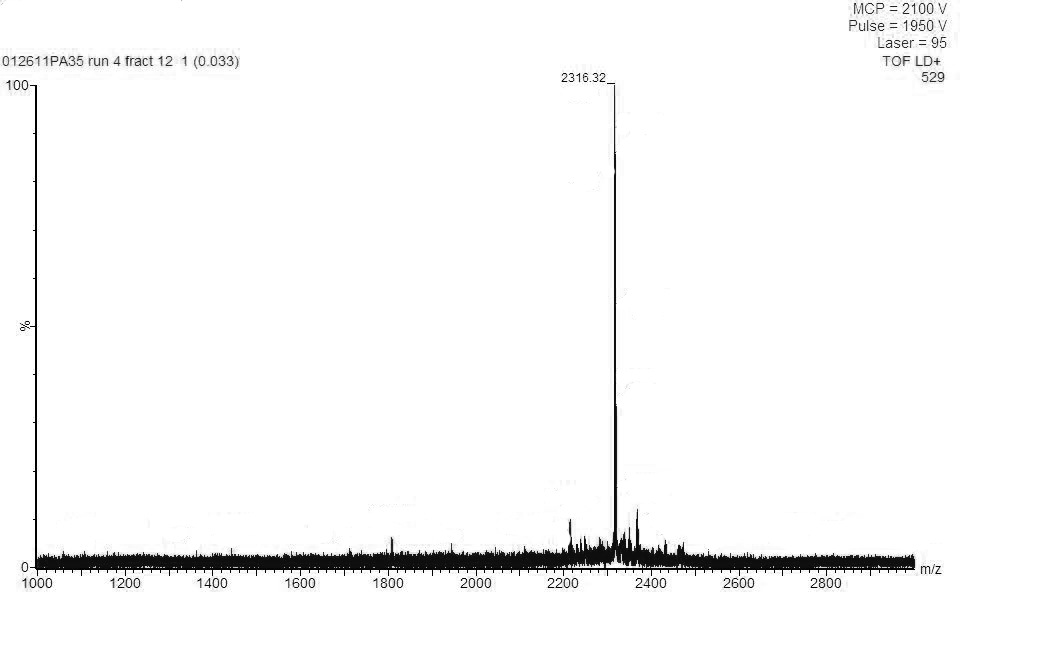


**Figure S4. Analysis of linear Tv1 peptide by MALDI-TOF mass spectrometry.** MALDI-TOF spectrum of Tv1 peptide using α-Cyano-4-hydroxycinnamic acid matrix


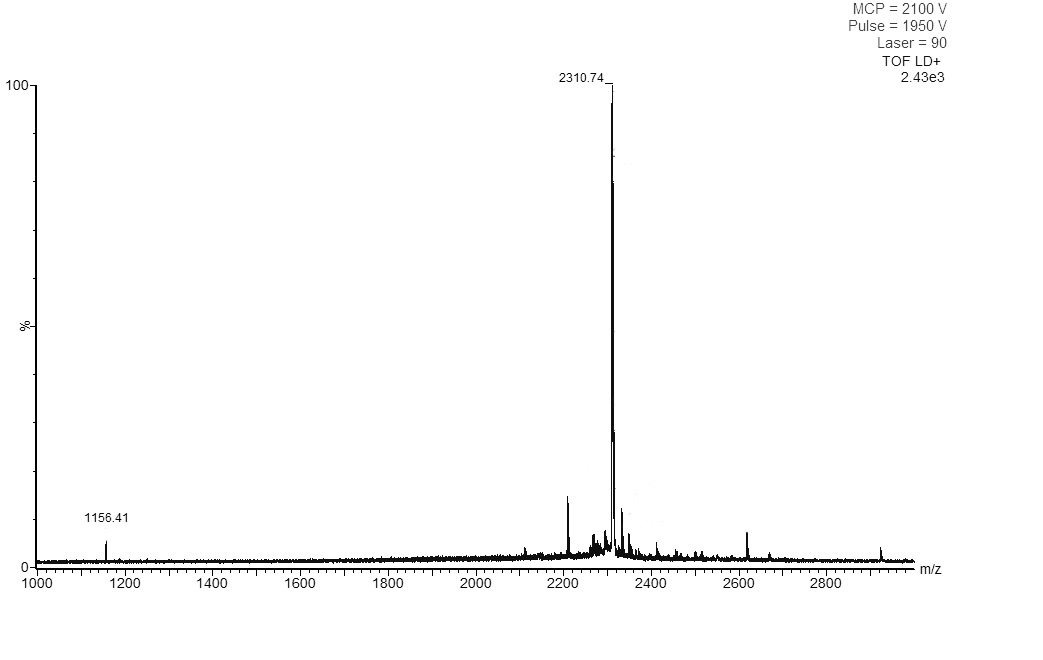
**Figure S5. Analysis of oxidized Tv1 peptide by MALDI-TOF mass spectrometry.** MALDI-TOF spectrum of Tv1 peptide using α-Cyano-4-hydroxycinnamic acid matrix


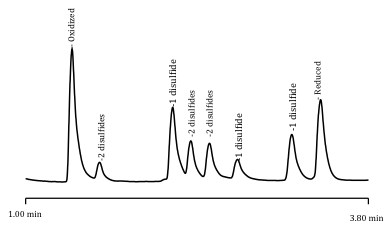


**Figure S6. RP-UHPLC analysis of partially reduced Tv1 peptide** on a UPLC (BEH 300 C18 1.7µm, Waters Corporation, Milford, MA, USA) column, a linear gradient of 0-75% buffer B (80% acetonitrile, 0.1% TFA) in buffer A (0.1% TFA) over 6 minutes and peaks were assigned by their degree of reduction. Labels indicate how many disulfides are present.

**
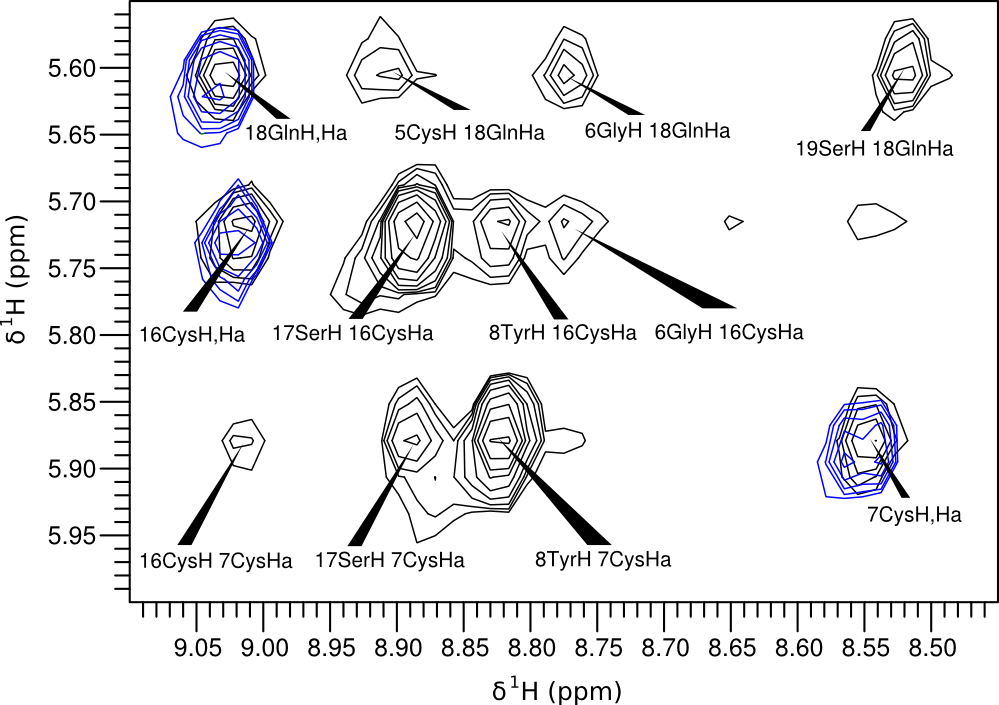
**

**Figure S7. NOE contacts confirming the C7-C16 disulfide bond.** An overlay of the H^N^H^α^ fingerprint region of the NOESY (in black) and TOCSY (in blue) spectra shows NOE crosspeaks linking Cys 16 and Cys 7 as well as contacts in the residues flanking the C7-C16 disulfide bond (C7-S17, Y8-C16, G6-C16, G6-N18).

**
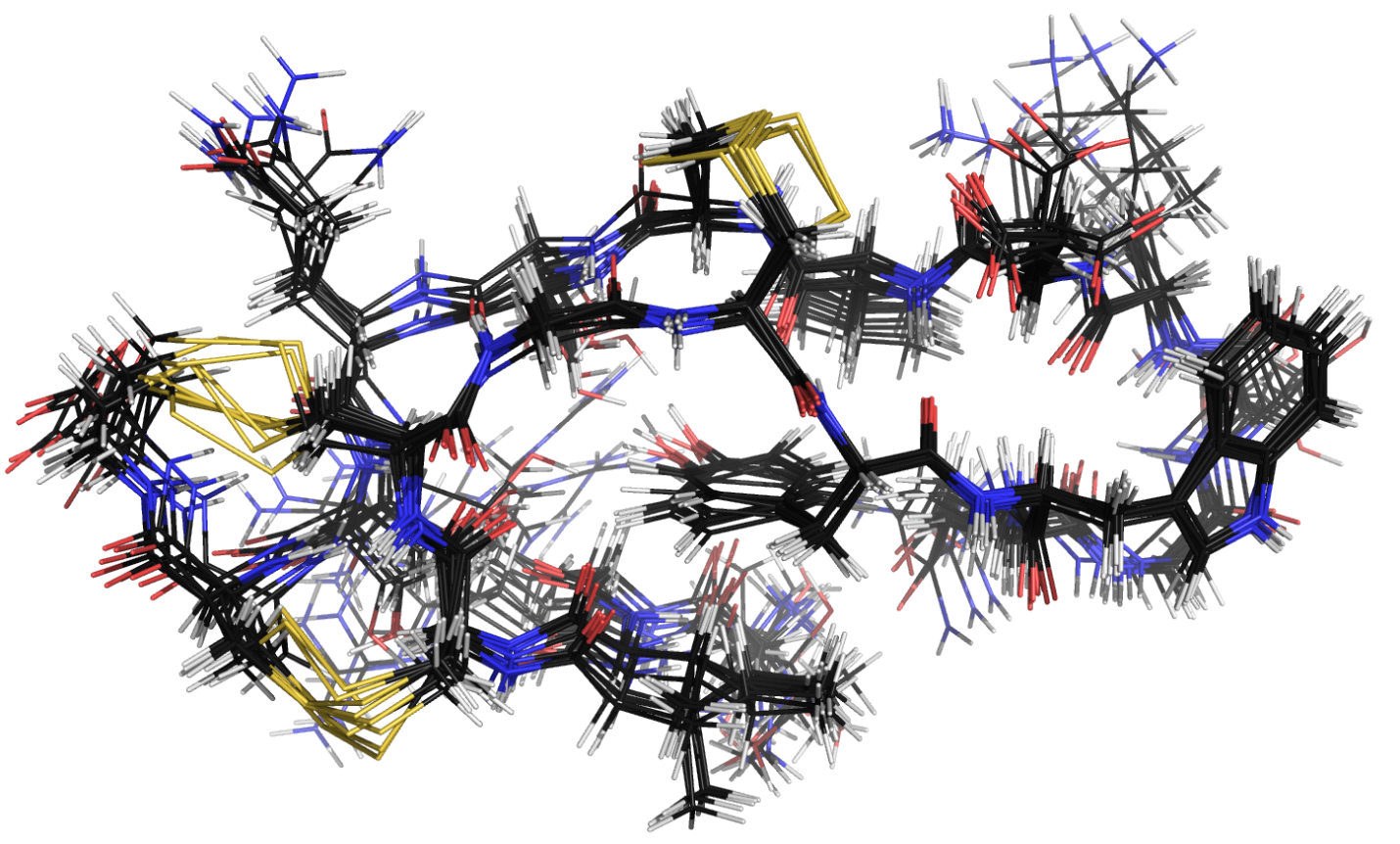
**

**Figure S8. Bundle of the 10 lowest energy structures of Tv1 after explicit water refinement.** The 10 lowest energy structures are shown in stick representation, displaying the tight convergence found in the final structural bundle.

**
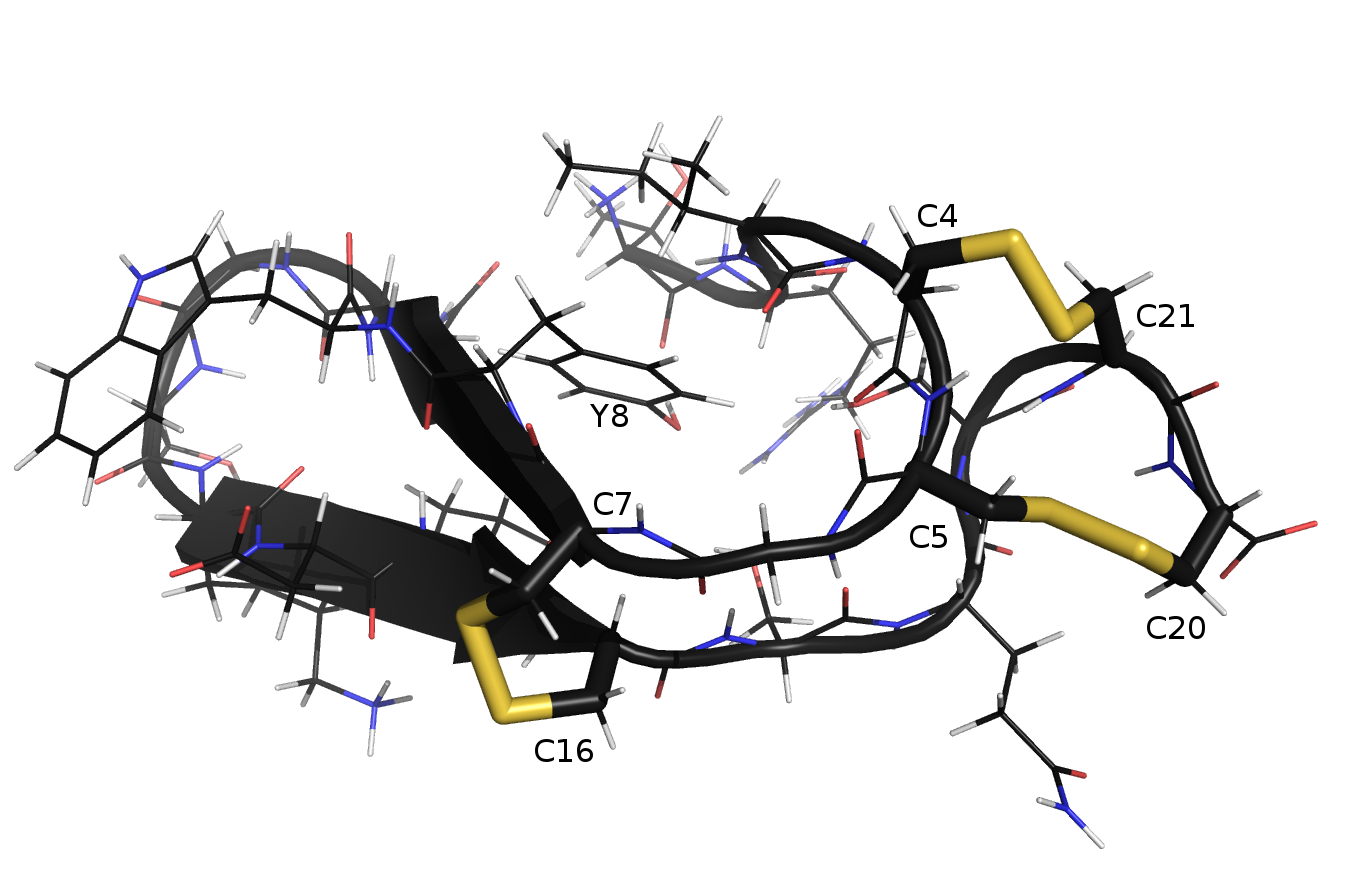
**

**Figure S9. Structure of Tv1.** An overlay of a cartoon representation and a stick model of the lowest-energy structure of Tv1 shows the β-sheet character of the peptide and reveals the important role of the Tyr 8 side chain in the formation of a small hydrophobic core.

***Supplementary Tables***

**Table S1.** Predicted and observed of b and y ions of differentially alkylated peptides by auto and targeted MS/MS analysis

| **Sequence** | **Ions** | **Unmodified peptide (m/z)** | **m/z**  **(thr.)** | **m/z**  **(obs.)** | **Difference** | **Modifications** |
| --- | --- | --- | --- | --- | --- | --- |
| **MS/MS for precursor ion-m/z 932.026 (+3) of parent ion 2793.0552** | | | | | | |
| TRIC | b4 | 474.2493 | 599.2902 | 599.297 | 125.0414 | NEM |
| TRICC | b5 | 577.2585 | 759.3197 | 759.3276 | 125.0414+57.0198 | NEM+IAM |
| C | y1 | 122.0270 | 179.048 | 179.0485 | 57.021 | IAM |
| CC | y2 | 225.0362 | 407.1048 | 407.1054 | 57.021+125.0476 | IAM+NEM |
| CCSQSC | y6 | 630.1680 | 869.2579 | 869.2586 | 57.021+125.0476+125.0479 | IAM+2NEM |
| **MS/MS for precursor ion-m/z 977.376 (+3) of parent ion 2929.1027** | | | | | | |
| TRICCG | b6 | 634.2800 | 884.3632 | 884.3753 | 250.0832 | 2NEM |
| TRICCGC | b7 | 737.2891 | 1044.4083 | 1044.406 | 307.1192(250.0831+57.036) | 2NEM+IAM |
| C | y1 | 122.0270 | 247.0747 | 247.0747 | 125.0477 | NEM |
| CC | y2 | 225.0362 | 475.1292 | 475.1316 | 250.093 | 2NEM |
| CCSQSC | y6 | 630.1680 | 937.2707 | 937.2849 | 57.0188+250.0859 | IAM+2NEM |

**Table S2.** Chemical shift assignments of Tv1 (in ppm)

|  | **H** | **H^α^** | **H^β^** | **H^γ^** | **C^α^** | **C^β^** | **C^γ^** |  |
| --- | --- | --- | --- | --- | --- | --- | --- | --- |
| Thr 1 |  | 4.07 | 3.63 | 1.26 | 70.18 | 65.06 | 21.4 |  |
| Arg 2 | 7.14 | 4.44 | 1.86  1.67 | 1.67  1.59 | 55.49 | 31.6 | 26.81 | H^δ^ 3.19, C^δ^ 43.42 |
| Ile 3 | 8.53 | 3.94 | 1.88 | 0.71  1.09  1.32 | 62.62 | 37.66 | 13.11  27.11 | H^δ^ 0.77, C^δ^ 17.40 |
| Cys 4 | 7.48 | 4.87 | 3.75  2.75 |  | 53.89 | 39.66 |  |  |
| Cys 5 | 8.91 | 5.45 | 4.30  2.58 |  | 55.11 | 48.6 |  |  |
| Gly 6 | 8.78 | 4.10  4.50 |  |  | 44.99 |  |  |  |
| Cys 7 | 8.55 | 5.89 | 3.04  2.69 |  | 55.23 | 49.51 |  |  |
| Tyr 8 | 8.82 | 4.97 | 3.04 |  |  | 41.28 |  | H^δ,*^ 7.01, H^ε,*^ 6.74 |
| Trp 9 | 8.93 | 5.2 | 3.33  3.25 |  | 54.38 | 30.5 |  | H^δ1^ 7.22, H^ε1^ 10.26, H^ε3^ 7.69, H^ζ2^ 7.27, H^ζ3^ 6.96, H^η2^ 7.04 |
| Asn 10 | 7.91 | 5.21 | 2.46  2.89 |  |  | 39.19 |  |  |
| Gly 11 | 5.47 | 4.51,3.51 |  |  | 45.1 |  |  |  |
| Ser 12 | 7.87 | 4.33 | 3.62 |  | 59.24 | 61.77 |  |  |
| Lys 13 | 7.59 | 4.34 | 1.57  1.71 | 1.17  0.90 | 55.28 | 34.88 | 22.89 | H^δa^ 1.18, H^εa^ 2.86, C^ε^ 41.57 |
| Asp 14 | 8.56 | 5.2 | 2.55  2.40 |  |  | 41.67 |  |  |
| Val 15 | 9.22 | 4.06 | 1.44 | 0.86  0.68 | 61.86 | 32.59 | 21.08,21.06 |  |
| Cys 16 | 9.02 | 5.73 | 2.97 |  | 55.22 | 47.32 |  |  |
| Ser 17 | 8.89 | 4.7 | 3.95  3.86 |  | 56.98 | 65.24 |  |  |
| Gln 18 | 9.03 | 5.62 | 2.40  1.96 | 2.39  2.31 | 55.18 | 29.85 | 33.79 |  |
| Ser 19 | 8.52 | 4.36 | 4.14  4.00 |  | 61.52 | 63.4 |  |  |
| Cys 20 | 7.56 | 4.52 | 3.34  2.86 |  | 55.45 | 39.66 |  |  |

**Table S3. Structural statistics for the final 10 models of Tv1**

**Quantity** **Value**

Total unambiguous distance restraints 780

Intra residual 556

Sequential (| *i* – *j* | = 1) 73

Medium (2 ≤ | *i* – *j* | ≤ 4) 39

Long range 112

NOE violations in all models

> 0.5 Å 0

> 0.3 Å 9

> 0.1 Å 211

RMSD from the average atomic coordinates (Å)

Backbone

Most ordered region (residues 4-18) 0.31 ± 0.10

All residues 0.42 ± 0.21

All Atoms

Most ordered region (residues 4-18) 0.59 ± 0.29

All residues 0.74 ± 0.43

Deviations from idealized covalent geometry

Bond (Å) 0.0055 ± 0.0003

Angles (°) 0.72 ± 0.045

Improper dihedrals (°) 1.94 ± 0.14

Ramachandran analysis (%)

Residues in most favored regions 61.2

Residues in additionally allowed regions 32.9

Residues in generously allowed regions 5.9

Residues in disallowed Regions 0.0

**Table S4. Sample raw data of Tv1 bioactivity in polychaete worms**

| **Time** | **Tv1(20µM)** | **Tv1(20µM)** | **NSS** |  |  |  |  |  |  |  |  |  |  |  |  |
| --- | --- | --- | --- | --- | --- | --- | --- | --- | --- | --- | --- | --- | --- | --- | --- |
| 00:10 | 3 | 3 | 3 | 02:40 | 1 | 1 | 3 | 05:10 | 1 | 1 | 3 | 07:40 | 1 | 1 | 3 |
| 00:20 | 3 | 3 | 3 | 02:50 | 2 | 1 | 3 | 05:20 | 1 | 1 | 3 | 07:50 | 1 | 1 | 3 |
| 00:30 | 1 | 3 | 3 | 03:00 | 1 | 1 | 3 | 05:30 | 1 | 1 | 3 | 08:00 | 1 | 1 | 3 |
| 00:40 | 1 | 3 | 3 | 03:10 | 1 | 1 | 3 | 05:40 | 1 | 1 | 3 | 08:10 | 2 | 1 | 3 |
| 00:50 | 1 | 3 | 3 | 03:20 | 1 | 1 | 3 | 05:50 | 1 | 1 | 3 | 08:20 | 2 | 1 | 3 |
| 01:00 | 1 | 1 | 3 | 03:30 | 1 | 1 | 3 | 06:00 | 1 | 1 | 3 | 08:30 | 2 | 1 | 3 |
| 01:10 | 1 | 1 | 3 | 03:40 | 2 | 1 | 3 | 06:10 | 1 | 1 | 3 | 08:40 | 2 | 1 | 3 |
| 01:20 | 1 | 1 | 3 | 03:50 | 1 | 1 | 3 | 06:20 | 1 | 2 | 3 | 08:50 | 1 | 1 | 3 |
| 01:30 | 1 | 2 | 3 | 04:00 | 2 | 1 | 3 | 06:30 | 1 | 1 | 3 | 09:00 | 1 | 1 | 3 |
| 01:40 | 1 | 1 | 3 | 04:10 | 1 | 1 | 3 | 06:40 | 2 | 1 | 3 | 09:10 | 1 | 1 | 3 |
| 01:50 | 1 | 1 | 3 | 04:20 | 2 | 1 | 3 | 06:50 | 2 | 1 | 3 | 09:20 | 1 | 1 | 3 |
| 02:00 | 1 | 1 | 3 | 04:30 | 1 | 1 | 3 | 07:00 | 2 | 1 | 3 | 09:30 | 1 | 1 | 3 |
| 02:10 | 1 | 1 | 3 | 04:40 | 2 | 1 | 3 | 07:10 | 2 | 2 | 3 | 09:40 | 1 | 1 | 3 |
| 02:20 | 2 | 1 | 3 | 04:50 | 1 | 2 | 3 | 07:20 | 1 | 2 | 3 | 09:50 | 1 | 1 | 3 |
| 02:30 | 2 | 1 | 3 | 05:00 | 1 | 1 | 3 | 07:30 | 1 | 2 | 3 | 10:00 | 2 | 1 | 3 |

**Reference**

1. Chalkley RJ, Brinkworth CS, Burlingame AL (2006) Side-chain fragmentation of alkylated cysteine residues in electron capture dissociation mass spectrometry. J Am Soc Mass Spectrom 17: 1271–1274.
